# Improved diagnostic stewardship in carbapenem-resistant Enterobacterales gene detection helps in early initiation of targeted therapy

**DOI:** 10.1099/jmm.0.002029

**Published:** 2025-06-20

**Authors:** Partha Guchhait, Nairita Choudhuri, Bhaskar Narayan Chaudhuri, Tanni Datta, Arup Kumar Dawn, Pallab Das, Susmriti Dalui, Satadal Das

**Affiliations:** 1 Department of Microbiology and Molecular Biology, Peerless Hospitex Hospital and Research Centre Limited, Kolkata, India; 2 Department of Microbiology, Vellore Institute of Technology, Vellore, India; 3 Department of Biotechnology, Utkal University, Bhubaneswar, Odisha, India; 4 Department of Microbiology, Chittaranjan National Cancer Institute, Kolkata, India; 5 Department of Microbiology, Tata Medical Centre, Kolkata, India

**Keywords:** carbapenem-resistant *Enterobacterales* (CRE), diagnostic stewardship, EDTA carbapenem inactivation method (eCIM), modified carbapenem inactivation method (mCIM), New Delhi metallo-*β*-lactamase (NDM), OXA-48 like, RT-PCR, synergy test, Vitek 2

## Abstract

**Introduction.** Antimicrobial resistance (AMR) is an escalating global health crisis, leading to ~700,000 deaths annually. Without significant containment efforts, this number could surge to 10 million by 2050. Carbapenem-resistant organisms, particularly carbapenem-resistant *Enterobacterales* (CRE), *Pseudomonas aeruginosa*, and *Acinetobacter baumannii*, present a critical challenge due to their ability to evade potent carbapenem antibiotics.

**Hypothesis and Aim.** This study aimed to determine the prevalence of CRE among 1,317 culture-positive patients and to assess the impact of advanced diagnostic techniques, such as RT-PCR, modified carbapenem inactivation method (mCIM), EDTA carbapenem inactivation method (eCIM) and Vitek susceptibility testing, on improving diagnostic stewardship and patient outcomes.

**Methodology.** A retrospective cross-sectional study was conducted at Peerless Hospitex Hospital and Research Centre Limited, Kolkata, from June 2023 to May 2024. CRE isolates were identified from various clinical samples and subjected to phenotypic (Vitek 2, mCIM and eCIM) and genotypic real time polymerase chain reaction (RT-PCR) testing for carbapenemase genes. Data on demographics, specimen types, bacterial isolates, comorbidities, etc. were analysed.

**Results.** Out of 20,129 inpatient samples sent for culture during this 1-year period, 3,124 (15.51%) had culture-proven infections. A total of 1,317 *Enterobacterales* isolates were processed for carbapenem resistance (CR) detection PCR, with 354 (26.88%) identified as CRE. *Klebsiella pneumoniae* was the predominant isolate (60.17%), followed by *Escherichia coli* (26.55%). New Delhi metallo-*β*-lactamase (MBL) (NDM) and OXA-48-like co-production (33.75%) were most commonly seen, followed by NDM gene alone (32.50%). The concordance between phenotypic susceptibility and genotypic PCR method for CRE was 85.88%. Targeted antibiotic therapy could be initiated based on PCR results, in 70.90% of cases. Synergy test guided effective combination therapy of ceftazidime-avibactam and aztreonam for MBL-producing CRE isolates.

**Conclusion.** The study highlights a significant prevalence of CRE, particularly among older adults. Advanced diagnostic techniques improved diagnostic stewardship, allowing timely and accurate detection of CR. However, discrepancies between phenotypic and genotypic methods and the high cost of certain therapies are notable limitations. Enhanced infection control and early initiation of targeted therapy are crucial to combat AMR.

## Introduction

With an estimated annual 10 million deaths by 2050 from drug-resistant bacterial infections [1] and the highest burden of antimicrobial resistance (AMR)-associated deaths in South Asia [2], AMR containment is one of the major issues globally. Among diverse AMR-contributing factors, delay in the effective appropriate treatment is becoming a central reason for deaths from infections, principally those related to carbapenem-resistant organisms (CROs). Other major factors are excessive antibiotic use, particularly in BRICS countries (Brazil, Russia, India, China and South Africa), poor infection control and international travel. According to the latest report from the ICMR AMR surveillance network, 28%, 55% and 80% resistance to imipenem was found in *Escherichia coli*, *Klebsiella pneumoniae*, and *Acinetobacter baumannii*, respectively. Among these, carbapenem-resistant *Enterobacterales* (CRE), carbapenem-resistant *Pseudomonas aeruginosa* and carbapenem-resistant *A. baumannii* are of main concern, and currently, the World Health Organization (WHO) has classified them as critical or highest-priority pathogens on the global priority list [3]. In India alone, the use of carbapenems has increased by 45% over the past decade, contributing to the spread of CROs [4].

Among these, CRE represent a major concern. CRE are known for their ability to resist multiple classes of antibiotics, including carbapenems, which are often the last line of defence against serious infections. The prevalence of community-acquired CRE has been reported to range from 0% to 29.5% [5], and early screening for CRE colonization in patients is crucial for preventing or limiting outbreaks through effective isolation measures. Resistance to carbapenems in CRE is mediated by various mechanisms. The hydrolysis of carbapenems by beta-lactamases is the major resistance mechanism. Beta-lactamases are classified into four classes (A, B, C and D) based on their structure and function according to Ambler’s classification [6]. Class A carbapenemases include *K. pneumoniae* carbapenemase (KPC) and extended-spectrum beta-lactamases (ESBLs). Class B carbapenemases, such as New Delhi metallo-*β*-lactamase (MBL) (NDM) and Verona integron-encoded MBL (VIM), are metallo-beta-lactamases that require zinc for their activity and can break down most beta-lactams except aztreonam (ATM), often found on plasmids. Class D includes oxacillinase (OXA-48) enzymes, which also use serine but are less effective against carbapenems and are inhibited by avibactam; these are frequently found in *K. pneumoniae* and are similar to ESBLs [6,8]. Resistance mechanisms also include decreased permeability through modifications of the outer membrane, overexpression of efflux pumps and target site mutations that render carbapenems less effective [7]. Together, these mechanisms complicate the treatment of carbapenem-resistant infections.

Early and accurate identification of carbapenem resistance (CR) is essential for both guiding appropriate therapy and implementing robust infection control measures. There are two primary approaches to detecting CR: phenotypic and genotypic methods. Phenotypically, biochemical assays such as the Carba NP®, Blue Carba® and Carba® tests were used. Carba NP detects class A and class B carbapenemases with a sensitivity and specificity of >90% with rapid turnaround time (5 min–2 h); however, these methods fail to detect other resistance mechanisms such as porin loss and efflux pump. Class D carbapenemases and mucoid isolates yielded false negatives. It does not differentiate the class of carbapenemases also (Table 3B and 3C: Tests for Carbapenemase in *Enterobacterales*; CLSI, 2025 and Guidance on Diagnosis and Management of Carbapenem Resistant Gram-negative Infections; ICMR 2022). The carbapenem inactivation method [modified carbapenem inactivation method (mCIM) and EDTA carbapenem inactivation method (eCIM)] is an inexpensive CLSI-recommended method for routine detection of carbapenemases with high sensitivity (>90%) and specificity (>90%) for class A (KPC), class B (Imipenemase [IMP], Verona-integron- enconded- metallo-beta-lactamse [VIM] and New Delhi Metallo-beta-lactamase [NDM]) and class D (OXA-48 like) carbapenemases, although long turnaround time (18–24 h) and inability to detect class B when co-expressed with class A/D carbapenemases remain the limitations: Tests for Carbapenemase in *Enterobacterales*; CLSI, 2025). On the other hand, genotypic methods, including molecular techniques like PCR, identify specific carbapenemase genes responsible for resistance, with very high sensitivity and specificity of 92.2% and 99.6%, respectively [9].

Not many large-scale studies have been conducted in India, to know the regional variation of the prevalence of CRE and to assess the effectiveness of real-time open PCR in early initiation of targeted therapy in hospitalized patients. So, the primary aim of this study is to determine the prevalence rate of CRE among culture-positive patients by real-time PCR. This also includes analysing bacterial antibiotic-resistant genotypes in relation to demographic factors, specimen types, bacterial isolate patterns and comorbidities. The secondary objective is to assess whether implementing multimodal advanced diagnostic techniques, such as Real time Polymerase chain reaction (RT-PCR), mCIM, eCIM and Vitek susceptibility testing, can effectively enhance diagnostic stewardship and improve overall patient outcomes by facilitating timely and accurate detection of CR.

## Methods

A retrospective, cross-sectional study was carried out in the Department of Microbiology at Peerless Hospitex Hospital and Research Centre, Kolkata, from June 2023 to May 2024. The Gram-negative bacilli isolated during 1 year from In-patient department (wards and Intensive care unit areas were included in the study.

### Inclusion criteria

All CRE being isolated during the study period and the treating physician requested for molecular study for further management of patients were included in the study.

### Exclusion criteria

Carbapenem-resistant Gram-negative bacteria other than *Enterobacterales* and repeat samples from the same patients during the hospitalization period were excluded from the study.

### Data collection

Detailed information of 1,317 patients along with their comorbidities and recent medical history was extracted retrospectively from the Department of Microbiology between June 2023 and May 2024. All CRE-positive culture reports [from blood, urine, sputum, Broncho alveolar lavage (BAL) fluid, endotracheal (ET) suction, pus, wound swabs, cerebrospinal fluid and other body fluids] where the real-time PCR was performed were included in the analysis.

On day 1, specimens like urine (*n*=168), blood (*n*=30), ET secretion (*n*=25), sputum (*n*=31), tissue (*n*=5), sterile body fluids (*n*=75) and pus (*n*=20) were inoculated simultaneously on MacConkey, blood and chocolate agar. In addition, blood and body fluids were also loaded into the Bactec FX40 system (Becton Dickson) in parallel. A direct susceptibility test was put up if bottle gram stain from a positive blood culture bottle revealed Gram-negative bacilli. On day 2, Gram-negative bacilli were identified based on colony characteristics and biochemical reactions and subsequently processed for antimicrobial susceptibility using the Vitek 2 automated system (bioMerieux, Durham, NC) along with additional antibiotic discs/E strip including ceftazidime-avibactam (CZA) and ATM and interpreted as per CLSI, 2025 guidelines on day 3. On day 2, in parallel to Vitek, the same colony was processed further to determine carbapenemase resistance gene(s) by open system multiplex real-time PCR using TRUPCR® Carbapenem Resistance Detection Kit, which detects the most relevant carbapenemase genes (e.g. NDM, KPC, OXA-48 like, IMP and VIM), with results available within 2–3 h. Additionally, isolates were subjected to mCIM and eCIM in parallel to detect carbapenemase production and differentiate serine from metallo-beta-lactamase as per CLSI, 2025 guidelines. PCR results on day 2 afternoon help guide clinicians to narrow down to definitive antimicrobial therapy, at least 14–16 h before the Vitek susceptibility report, thus improving diagnostic and antimicrobial stewardship to a greater extent. On day 3, phenotypic Vitek susceptibility results (available after 16–18 h of PCR result) were interpreted as per MIC and Disc diffusion breakpoint tables of *Enterobacterales* (CLSI, 2025) and cross-checked with genotypic PCR and phenotypic (mCIM and eCIM) findings. A comprehensive report is thus generated, indicating the identified organism, resistance patterns, CZA and ATM synergy result and suggested antibiotics, enabling early initiation of targeted therapy based on the combined results. This systematic approach ensures prompt and accurate detection of CROs, facilitating timely and appropriate clinical interventions to manage AMR effectively.

### RT-PCR process

To perform the TRUPCR® assay, nucleic acid was extracted from an isolated colony using the QIAamp DNA Mini Kit (QIAGEN), and the elute was added to the prepared Master Mix and run on the real-time PCR machine according to the specified thermal cycling conditions. Positive, negative and internal controls were checked before interpreting the sample result. A positive result is indicated by the detection of fluorescence specific to each carbapenemase gene as per the manufacturer’s instruction for interpretation of results (as per TRUPCR Kit insert). If the internal control fails or no fluorescence is detected, it may suggest issues like sample contamination, poor extraction or assay errors.

### mCIM/eCIM test

To perform the mCIM and eCIM tests, a loopful of test bacteria was transferred from an overnight agar plate to two tubes, each containing 2 ml of trypticase soy broth (TSB), and vortexed the suspensions for 10–15 s. Then, a 10 µg meropenem disc was placed into each TSB tube using sterile forceps. To one of the tubes, 20 µl of 0.5 M EDTA was added to achieve a final concentration of 5 mM EDTA and labelled for the eCIM test. TSB-disc suspensions were incubated at 35 °C for 4 h. Before the incubation ended, a 0.5 McFarland suspension of *E. coli* ATCC 25922 was prepared and lawn cultured onto a Mueller–Hinton agar (MHA) plate. Meropenem discs from both TSB suspensions were removed, ensuring that excess liquid was removed and placed onto the inoculated MHA plate and incubated at 35–37 °C overnight. For the mCIM test, a positive result is indicated by a zone of inhibition measuring 6–15 mm or the presence of colonies within a 16–18 mm zone, while a negative result shows a zone of inhibition measuring 19 mm or greater. For the eCIM test, we compared the inhibition zone around the meropenem disc from the eCIM test to the mCIM test. If there is a ≥5 mm increase in the inhibition zone for eCIM compared with mCIM, it indicates MBL production, as EDTA inhibits the MBL activity, enlarging the inhibition zone. If there is a ≤4 mm increase, it indicates serine carbapenemase production, as EDTA does not affect these enzymes (Table 3C, CLSI, 2025). Both mCIM and eCIM have a high sensitivity and specificity (>95%) [10].

### Modified E test/disc method

CZA E test strip was placed on an MHA plate after the lawn culture of the test isolates. One ATM disc was placed 15 mm away from the sensitive breakpoint of CZA, i.e. 8 µg ml^−1^. After overnight incubation, a reading was taken to detect the presence or absence of synergy and interpreted as mentioned in ‘Guidance on Diagnosis and Management of Carbapenem Resistant Gram-negative Infections’, ICMR 2022. The inverse D zone suggests the presence of an MBL producer. The modified E test/disc method has also a high sensitivity and specificity (>95%) [11].

## Results

**Fig. 1.**
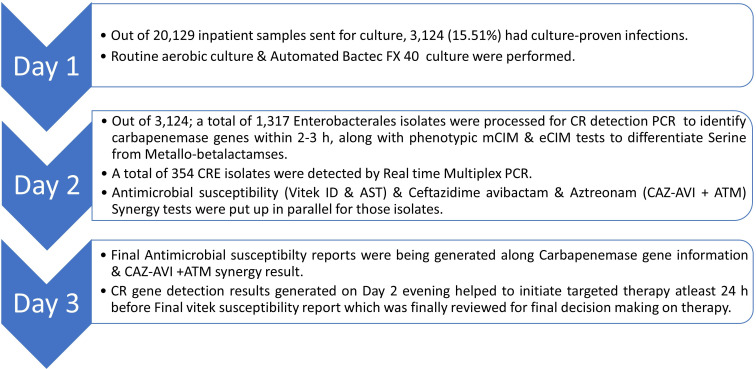
Details of the workflow in this study.

Out of 20,129 inpatient samples sent for culture during this 1-year period, 3,124 (15.51%) had culture-proven infections. A total of 1,317 *Enterobacterales* isolates were processed for CR detection PCR, with 354 (26.88%) identified as CRE. Out of 1,317 culture-positive infections, we were able to gather demographic details of 1,004 isolates, 320 of which were CRE. Details of the workflow are summarized in Fig. 1.

**Fig. 2.**
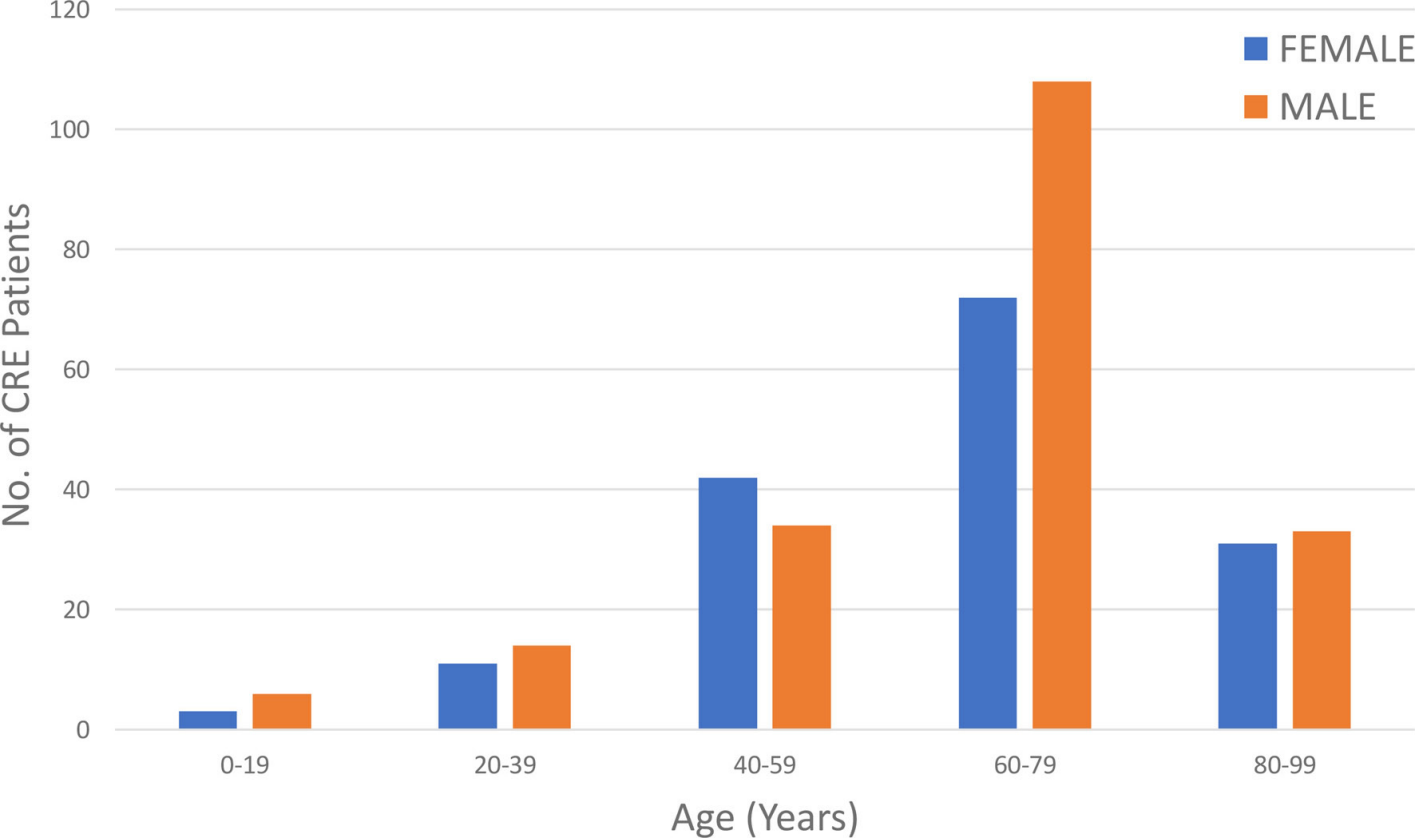
Age-wise gender distribution of CRE patients in the study population.

Age-wise distribution of the study population revealed that the highest prevalence of CRE was seen among the 60–79-year age group, accounting for 50.85% of the total cases, with a male predominance in this category (60% males vs. 40% females), shown in Fig. 2. The second most affected age group is 40–59 years, comprising 21.47% of the cases, with a higher proportion of females (55.26%) compared with males (44.74%). Overall, males (54.80%) are more frequently affected than females (44.92%).

**Fig. 3.**
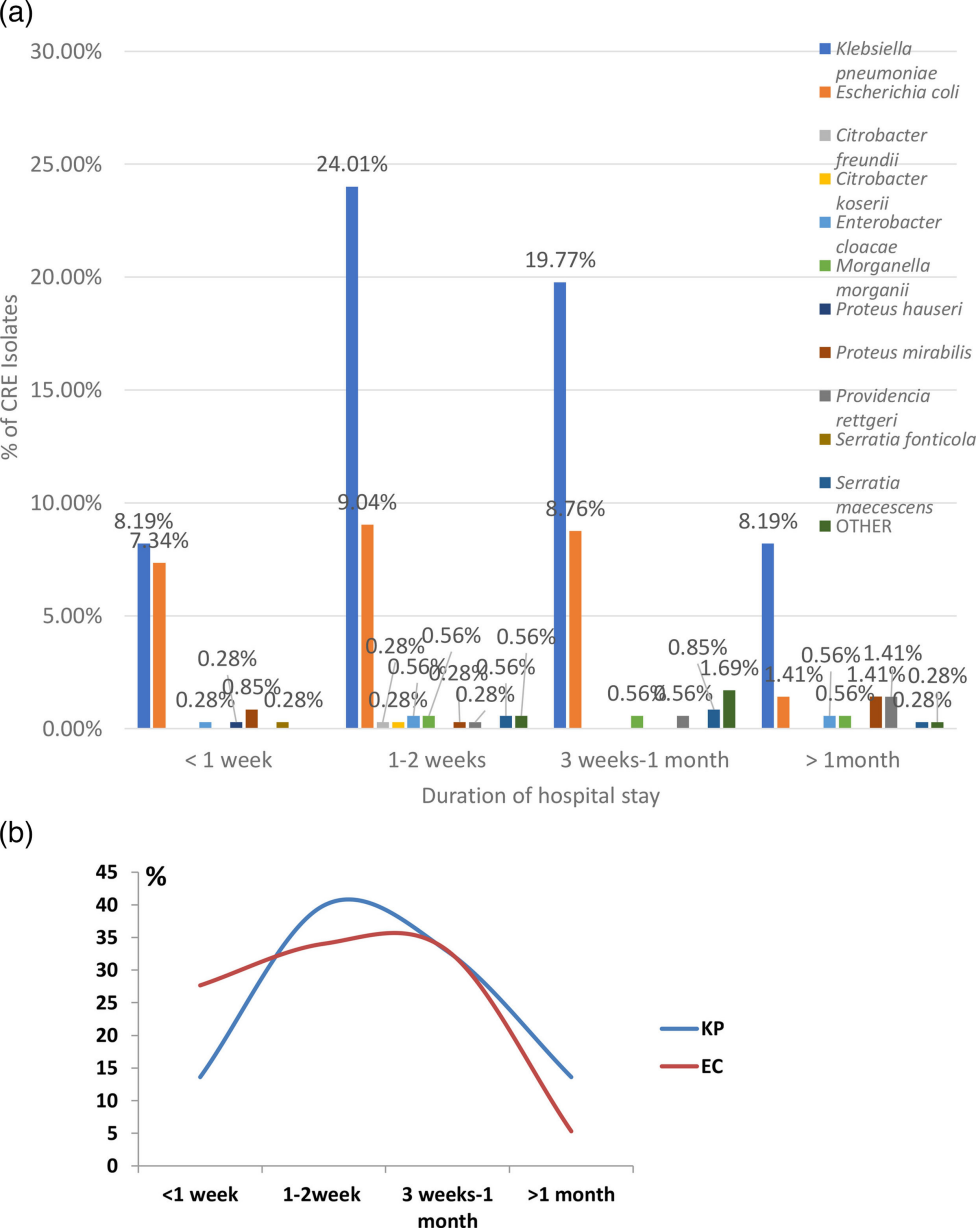
(a) Duration of hospital stay of CRE patients in relation to the bacterial isolates obtained from clinical samples; others include *Klebsiella aerogenes*, *Klebsiella oxytoca*, *Proteus vulgaris* and *Pantoea species*. (b) Comparison of hospital stays between CRE-positive *K. pneumoniae* (KP) and *E. coli* (EC)-positive cases. The differences in <1 week (*P* value 0.0031) and >1 month (*P* value 0.0329) are statistically significant, while differences in 1–2 weeks (*P* value 0.3298) and 3 weeks–1 month (*P* value 0.9836) are not statistically significant.

The majority of CRE patients (36.44%) had a hospital stay duration of 1–2 weeks, with *Klebsiella* species being the most common isolate in this category (65.89% of cases), depicted in Fig. 3(a, b). The second highest duration group, those staying between 3 weeks and 1 month, accounted for 32.20% of the cases, again dominated by *Klebsiella* species (61.40% of cases). Extended hospital stays (more than 1 month) were less common, representing 14.12% of the total cases, with *Klebsiella* remaining the predominant isolate (58% of cases).

**Fig. 4.**
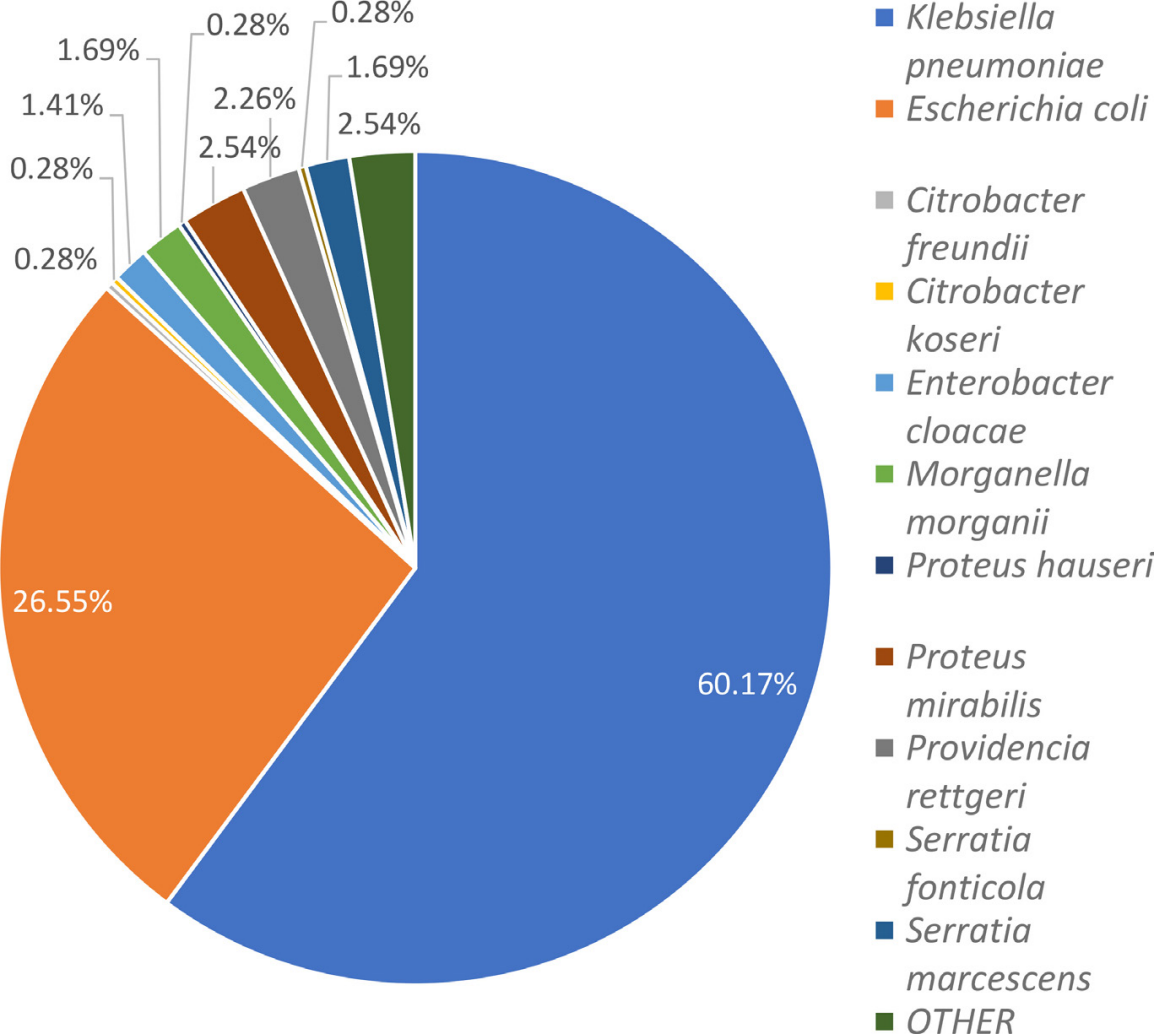
Distribution of different *Enterobacterales* species obtained from clinical samples; others include *Klebsiella aerogenes*, *Klebsiella oxytoca*, *Proteus vulgaris* and *Pantoea species*.

*K. pneumoniae* was the most prevalent species, found in 60.17% of the cases, followed by *E. coli* (26.55%) and other *Enterobacterales* species, such as *Morganella morganii* (1.69%), *Proteus* spp. (2.82%), *Citrobacter* spp. (0.56%), *Providencia rettgeri* (2.26%), *Serratia* spp. (1.98%), etc. (Fig. 4).

**Fig. 5.**
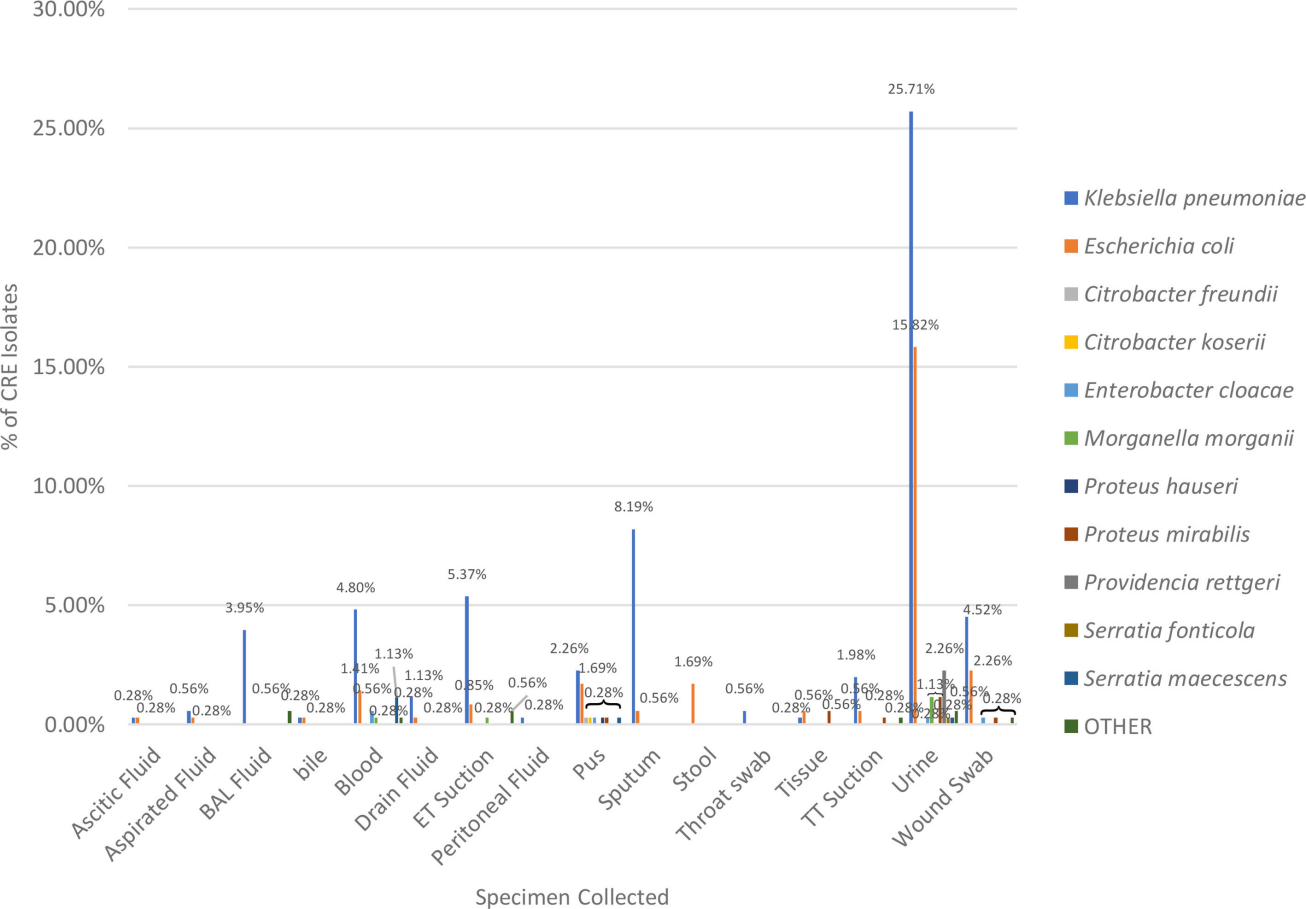
Sample-wise distribution of CRE obtained from the study population.

CRE is most commonly isolated from urine samples (47.46%), followed by sputum (8.76%), blood (8.47%), wound swab (7.63%) and other samples with lower frequency like ET aspirate (7.06%), pus (5.65%) and BAL fluid (4.52%) (Fig. 5).

Additionally, we found that diabetes mellitus (DM) and hypertension (HTN) were the most common dual comorbidities found (18.36%) among CRE patients. Those with only HTN and DM constituted 12.43%–11.02% respectively, while those with DM, HTN and hypothyroidism represented 5.93%. Other comorbid conditions included respiratory diseases and Parkinsonism, albeit at lower frequencies as mentioned in Table 1.

**Table 1.** Details of comorbidities among CRE patients

Comorbidity	% of CRE patients
DM	11.02
DM, HTN	18.36
DM, HTN, hypothyroidism	5.93
DM, hypothyroidism	2.82
HTN	12.43
HTN, hyperthyroidism	5.37
Hypothyroidism	1.98
No comorbidity	34.75
Other comorbidities (7 cases of Parkinsonism; 3 cases of carcinoma; 9 cases of respiratory problems like Chronic obstructive pulmonary disease [COPD] and asthma; 7 cases of Chronic kidney disease [CKD])	7.34
**Grand total**	**100 (354**)

**Fig. 6.**
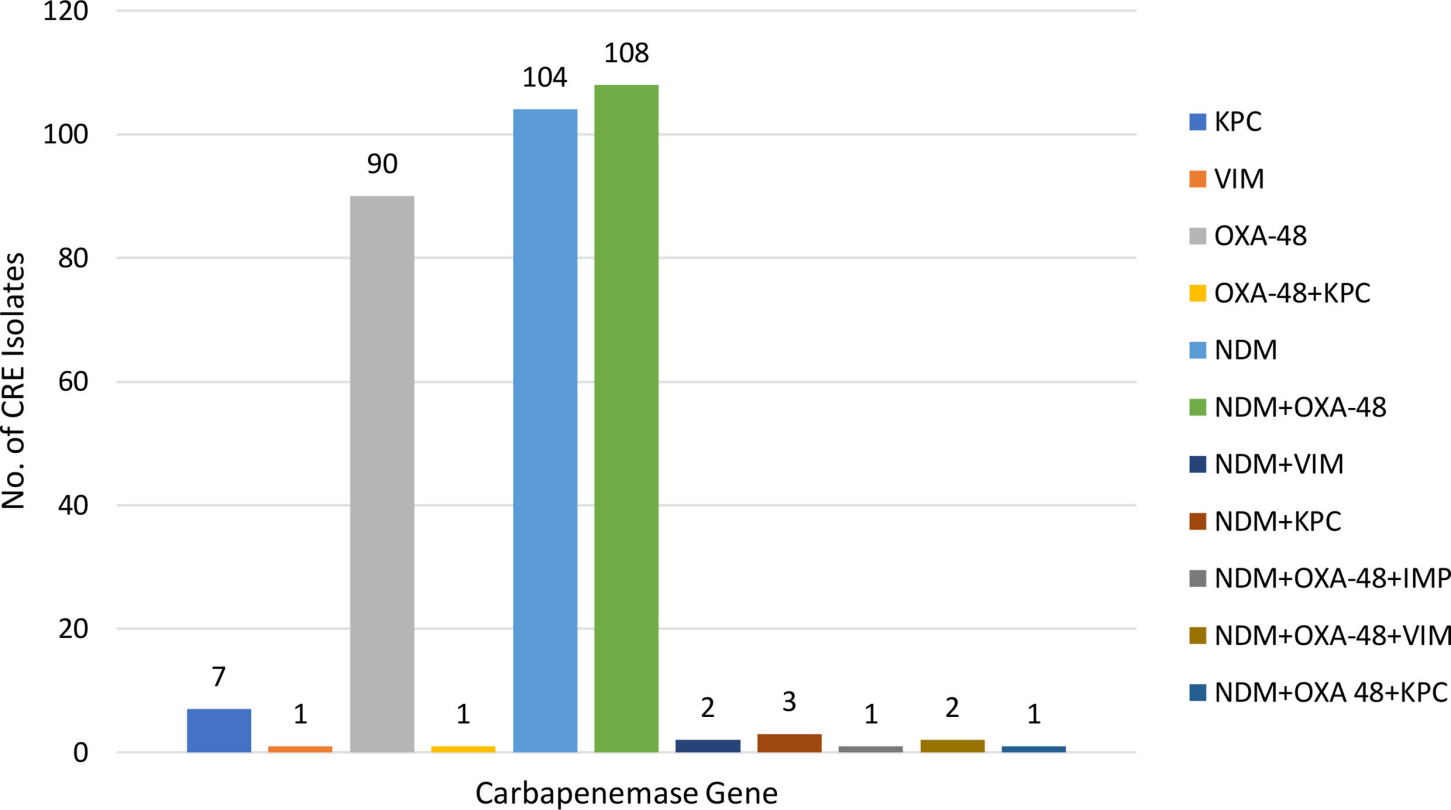
Prevalence of carbapenemase gene detected by open system RT-PCR from clinical isolates.

Among CRE isolates, 32.50% (104 out of 320) showed NDM gene alone, while 28.13% (90 out of 320) showed isolated OXA-48-like gene alone and 2.18% (7 out of 320) showed KPC gene as the inducer of resistance. A total of 33.75% (109 out of 320) of CRE isolates showed the presence of both NDM and OXA-48-like genes. Less common genes included KPC+NDM (0.94%), and others with lower prevalence such as VIM and IMP were seen alone or in combination (Fig. 6).

Genotypic results by CR detection PCR from bacterial isolates were compared with phenotypic CR reported by Vitek 2 compact system, using Vitek N405 card for *Enterobacterales*. There was a high concordance rate (85.88%) noted between phenotypic (Vitek 2 compact susceptibility report) and genotypic (real-time PCR report) detection results for CR detection. However, 9.60% of cases showed discordant results, where CR (at least one or more carbapenem showed resistant pattern) was detected in Vitek, with a negative genotypic result for carbapenemase genes by real-time multiplex PCR, and a small fraction (4.52%) showed non-CRE phenotype by Vitek but were genotypically positive for CRE. A detailed comparison between phenotypic (Vitek susceptibility report) and genotypic (CRE detection by real-time multiplex PCR) results is depicted in Table 2.

**Table 2.** Phenotypic susceptibility (Vitek) vs. genotypic detection (PCR): concordant and discordant results of CR between Vitek 2 compact susceptibility and real-time PCR among CRE isolates

Pattern of Vitek susceptibility results and real-time multiplex PCR reports	No. of reports (%)
Vitek showed CRE pattern and PCR also detects CR gene	304 (85.88)
Vitek showed non-CRE pattern but PCR detects CR gene	16 (4.52)
Vitek showed CRE pattern but PCR detects no CR gene	34 (9.60)
**Grand total**	**354**

MedCalc Software for comparison of proportions (version 23.1.7) was used to compare the results of phenotypic and genotypic methods.

Although statistically, the correlation between phenotypic and genotypic detection of CRE was highly significant (*P*<0.0001), false-positive and false-negative phenotypic results may coexist, which may alter the management of CRE patients.

In addition, PCR was able to identify 222 isolates with MBL genes (class B), while the rest were serine beta-lactamases. The mCIM/eCIM tests were performed simultaneously, showing an 85% concordance with PCR results for serine beta-lactamases, and 95% accuracy was seen for MBL genes. Although mCIM/eCIM is time consuming, less sensitive and specific compared with PCR, it has proven to be a useful phenotypic method for carbapenemase production, especially in resource-limited settings, and is recommended by CLSI also.

**Fig. 7.**
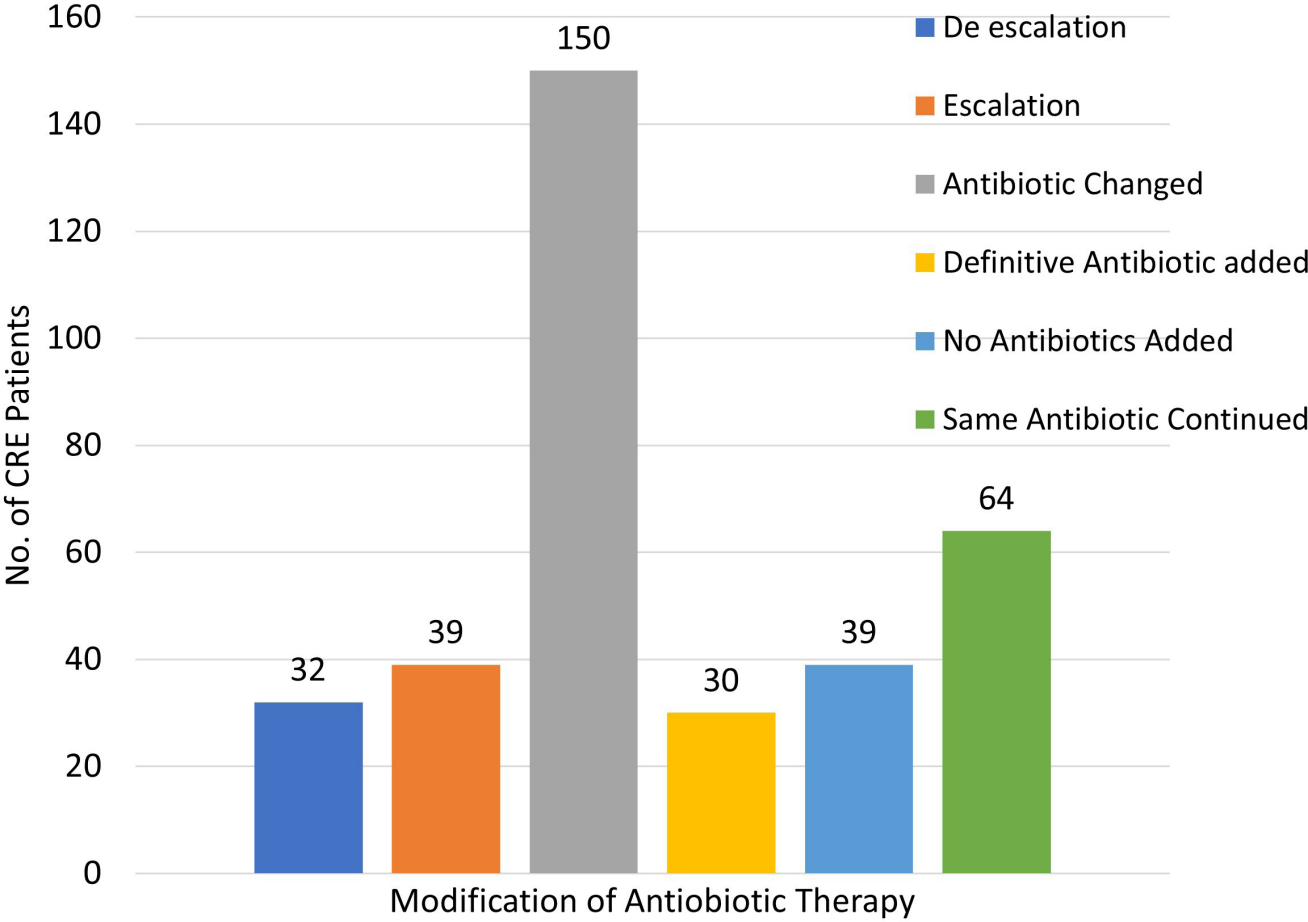
Antibiotic therapy modification as per CR detection PCR report.

The analysis of antibiotic therapy adjustment based on PCR result revealed that 42.37% of CRE patients had their empirical antibiotic regimen changed to definitive antibiotic therapy after obtaining the CRE detection PCR reports (on day 2) and Vitek report (on day 3), highlighting the importance of tailored therapy based on specific resistance patterns (Fig. 7). De-escalation occurred in 9.04% of cases, where therapy was stepped down to a less broad-spectrum antibiotic, reducing resistance and side effects. In contrast, in 11.02% of cases, escalation in therapy was noted, due to the identification of highly resistant pathogens requiring stronger or combination antibiotics and guarded prognosis of patients.

**Fig. 8.**
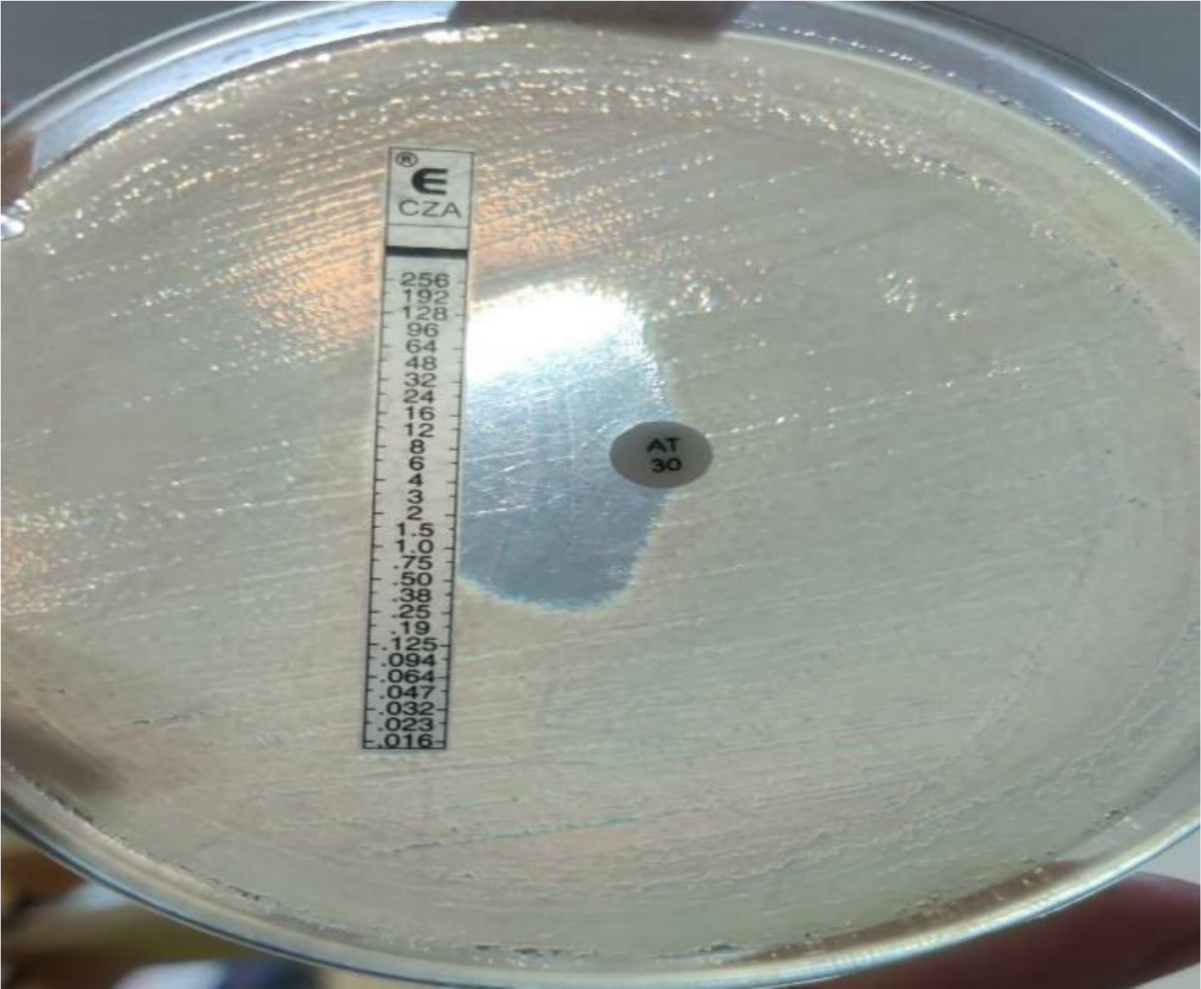
Modified E test/disc with CZA E test and ATM disc (30 µg) placed 15 mm apart: synergy demonstrated by inverse D.

For isolates carrying MBL gene(s) alone (NDM, VIM and IMP) or in combination, synergy tests were performed to determine the efficacy of combination therapy using the modified E test/disc method, as recommended by ICMR in its guidance on CRE detection, 2022. If the synergy test was found to be positive (in the form of inverse D zone of inhibition around ATM disc towards CZA E strip) (Fig. 8), a combination therapy with CZA and ATM was administered, unless cost constraints were a factor. In cases where the synergy test was negative, polymyxin B was used along with some sensitive antibiotic, which shows synergistic action. In our study, among the isolates that demonstrated synergy, the most common organism was * K. pneumoniae*, accounting for 63.56% of the total carbapenem-resistant *K. pneumoniae* cases (*n*=129), followed by *E. coli*, with 56.66% case synergism that could be elicited among total carbapenem-resistant *E. coli* isolates (*n*=60). The use of synergy tests helped tailor the treatment approach, optimizing the chances of successful outcomes for patients with MBL-producing infections.

## Discussion

The prevalence of culture-positive infections in our study was 15.51%, with a significant portion caused by *Enterobacterales*. The high rate of CRE infections (26.88%) among these cases underscores the growing threat of AMR and aligns with global trends. By analysing the clinical and microbiological data from these CRE patients, we seek to understand the distribution of CRE pathogens, the role of various carbapenem genes and the effectiveness of both phenotypic and genotypic methods in guiding appropriate antimicrobial therapy and improving diagnostic stewardship.

CRE prevalence varies globally, with notable patterns in different regions. In our study, CRE infections were most prevalent among older adults aged 60–79 years, with male predominance. The second most affected group was 40–59 years, with more females, and the lowest age group was 0–19 years. In Europe, most CRE cases occur in the 19–64 age groups, with a higher incidence in males compared with females [12]. Similarly, a study in South-West Nigeria found the highest proportion of CRE infections in the 40–54-year age group (35.9%) and the lowest in the 17–24-year age group (0.26%) [13]. In China, the average age of CRE patients is 62 years, and there is also a higher prevalence among males [14]. These demographic similarities and differences emphasize the need for region-specific strategies for managing CRE infections.

We observed that most CRE patients stayed in the hospital for 1–2 weeks, with *Klebsiella* species being the most common isolates. Similarly, in the study by Tamma *et al*. [15], the median duration of hospital stays for patients colonized with CRE was 13 days, with an interquartile range of 7–21 days. This metric reflected the time from the initial rectal swab for CRE until discharge, showing that patients colonized with CRE had a relatively short hospital stay on average. In another study by Sharma *et al*. [16], longer hospital stays were associated with an increased risk of CRE colonization. Specifically, patients who were colonized with CRE had a median hospital stay of 17 days, significantly longer than the 9 days for patients who were not colonized (*P*<0.001). This finding underscores that extended hospital stays can elevate the risk of acquiring CRE. This is also backed in the article by Adams *et al*. [17] which mentioned that patients with CRE infections faced significantly higher hospitalization costs, with an average increase of $206,664 (*P*<0.001), experienced longer hospital stays by 28.8 days (*P*<0.001) and had a higher risk of in-hospital mortality, with an adjusted odds ratio of 3.34 (95% CI, 1.82–6.12).

*K. pneumoniae* (60.17%) was the most prevalent pathogen isolated from our study population, emphasizing its role as a primary pathogen in healthcare-associated infections. *E. coli*, though less common, also highlighted the need for strict infection control. * K. pneumoniae* is consistently identified as the most common bacterial isolate in most studies of CRE infections both internationally [18,20] and in India [16,21, 22] followed by *E. coli* as the second most prevalent pathogen.

In our study, urine samples were the most significant source for CR bacterial isolates, indicating the urinary tract as a major infection site, with *K. pneumoniae* (54.17%), *E. coli* (33.33%), *Proteus* spp. (2.54%), *M. morganii* and *Serratia marcescens* (1.69%) being predominantly isolated from this specimen type. This is consistent with several studies such as Adesanya and Igwe, Prabhala *et al*. and Moghnieh *et al*. [13,22, 23] where urine was also the most common specimen source (40.7%, 28.4% and 31%, respectively). In contrast, Kotb *et al*. [24]] reported that CRE isolates were more frequently found in blood specimens.

Additionally, our study highlights DM and HTN as the most common dual comorbidities among CRE patients, indicating a higher vulnerability to infections, with 18.36% of patients having both conditions and 11.02% having only DM. This is somewhat similar to Moghnieh *et al*. [23], where cardiovascular disease and DM were the most common comorbidities, affecting 51.6% and 43.2% of patients, respectively, and a substantial portion of the patients had chronic wounds and had undergone recent surgical procedures. In addition, Aiesh *et al*. [18] showed that malignancy was a notable comorbidity in 36.7% of CRE patients and a significant number of CRE cases were identified in the emergency department (18.3%). Additionally, the study by Alexander *et al*. [25] found that a substantial proportion of patients had severe underlying conditions: 48.6% had been hospitalized for over 13 days at the time of CRE infection, 32.8% had chronic renal insufficiency and 26.2% were immune-compromised. The study also revealed that these patients often presented with severe sepsis (32.8%) or septic shock, which greatly contributed to the high 28-day mortality rate of 28.1% and poor clinical outcomes despite receiving the best available therapy. Managing these underlying conditions is crucial to mitigate CRE infection risks.

The detection of CRE is challenging due to the heterogeneous expression of CRE-resistant genes and diverse mechanisms, leading to resistance in bacteria [26], e.g. porin structural changes, efflux pump overactivation, changing binding sites and non-expression of carbapenemase gene phenotypically due to the loss of plasmid and carbapenemase genes that are not included in the routine PCR panel, such as Guiana-extended-spectrum, *S. marcescens* enzymes, imipenem hydrolysing and non-metallo enzyme carbapenemase class A, which are rare carbapenemases [27,28]. Phenotypic tests cannot reliably detect all types of resistance, and false-negative results may occur, which may lead to adverse treatment outcomes. Our results also showed false-negative (4.52%) and false-positive (9.60%) phenotypic results, which could be due to the above factors. However, as we could not reliably rule out the possibility of the existence of other carbapenemase genes, which are not included in our multiplex PCR system and other mechanisms of CR, etc.; therefore, false-positive and false-negative results may be relative and may not be an absolute representation of the real scenario. So, the authors recommend reviewing both genotypic and phenotypic results for final decision-making on definitive therapy.

The multilayered Coronavirus disease of 2019 (COVID-19) leads to a long-term uncertain effect on CRE by decreasing resistant gene transmission due to the preventive measures taken, while overuse of antibiotics for fear of acquiring secondary infections leads to the florid appearance of resistant genes. During COVID-19, the VIM gene was transmitted in lesser number, while OXA-48 genes were unchanged in the isolates, unlike the NDM gene which was on the rise. In general, NDM- and OXA-48-resistant genes were predominantly found in most of the reports from India. Our study revealed that 32.5% of CRE isolates carried the NDM gene alone, while 28.1% had the OXA-48 gene alone, and 33.8% of isolates had both NDM and OXA-48 genes. This gene distribution contrasts with the study by Joshi *et al*. [21], where the OXA-48 gene was predominant (84.5%) and NDM was the second most common gene (58.6%). A study by Taha *et al*. [17,20] also reported OXA-48 as the dominant gene (82.2%), with a lower prevalence of NDM (6.1%). In the study by Prabhala *et al*. [22], NDM was the most prevalent (33.68%), with a considerable presence of both NDM and OXA-48 (32.63%), aligning more closely with our findings but with a higher prevalence of NDM compared with OXA-48. El Defrawy *et al*. [19] highlighted NDM as the most common gene (84%) but found OXA-48 and KPC to be much less prevalent. Our study’s balanced prevalence of NDM and OXA-48, alongside the presence of KPC and less frequent VIM and IMP genes, suggests a relatively diverse CRE gene profile compared with the more dominant or singular gene profiles observed in other studies. Moreover, the high concordance rate (85.88%) between our phenotypic susceptibility and genotypic detection methods validates the reliability of our test results. Based on these reports, adjusting antibiotic therapy was essential. Most of our patients had their empirical therapy changed to definitive treatment within an hour following the CR gene detection report, thus reflecting the utility of rapid diagnostics. De-escalation reduced broad-spectrum antibiotic use, while escalation addressed the need for wide coverage encompassing highly resistant pathogens. The treatment of CRE infections presents significant challenges due to the broad and variable resistance conferred by different carbapenem enzymes. Moreover, in addition to *β*-lactams, CRE often show resistance to unrelated drug classes like aminoglycosides and fluoroquinolones, complicating treatment further, as traditional susceptibility tests might not capture all resistance mechanisms (explaining our discordant results). Currently, there are no large-scale randomized controlled trials for CRE treatment, so guidelines are primarily based on case reports, case series and smaller studies, which have limitations [29]. As a result, treatment recommendations suggest consulting infectious disease experts and using susceptibility results to guide therapy, which is primarily what we have done.

Although considering CR detection PCR in routine culture sensitivity follow-up is challenging in resource-limited settings, timely detection of CRE by real-time multiplex PCR directly from viable micro-organisms (using open system as closed system kits are expensive) not only helps clinicians to narrow down to targeted therapy much earlier for critical patients, but it may also significantly reduce morbidity, antibiotic consumption, cost and average length of stay in hospital. So, the authors recommend including CR detection PCR in routine workflow for inpatient culture isolates.

Regarding treatment, combining ATM with CZA provides a viable treatment option for ‘MBL infections’. This combination therapy should not be used empirically; rather, it can be used as a salvage therapy if susceptibility testing and synergy results are available. In our study, among the isolates that demonstrated synergy, the most common organism was *K. pneumoniae*, accounting for 63.56% of the total carbapenem-resistant *K. pneumoniae* cases (*n*=129), followed by *E. coli*, with 56.66% case synergism that could be elicited among the total carbapenem-resistant *E. coli* isolates (*n*=60). The use of synergy tests helped tailor the treatment approach, optimizing the chances of successful outcomes for patients with MBL-producing infections.

## Conclusion

Our findings emphasize the predominance of *K. pneumoniae* as the primary CRE pathogen and highlight the significant role of NDM and OXA-48-like genes in CR. The integration of phenotypic and genotypic methods, particularly the use of RT-PCR, has proven invaluable in guiding timely and accurate antimicrobial therapy, enhancing both diagnostic and antimicrobial stewardship and thus improving patient care and outcomes.

### Limitation

However, our study had several limitations. The retrospective design may have introduced selection bias, and the reliance on a single hospital’s data limits the generalization of our findings. Additionally, the exclusion of non-*Enterobacterales* CR isolates and few repeat samples may have impacted the comprehensiveness of the results. Future research should consider a larger, multi-centric approach to validate our findings and further explore the epidemiology and management of CRE infections.

## Abbreviations

AMR: antimicrobial resistance
ATM: aztreonam
COVID-19: coronavirus disease of 2019
CR: carbapenem resistance
CRE: carbapenem-resistant *Enterobacterales*
CROs: carbapenem-resistant organisms
CZA: ceftazidime-avibactam
DM: diabetes mellitus
eCIM: EDTA carbapenem inactivation method
ESBLs: extended-spectrum beta-lactamases
ET: endotracheal
HTN: hypertension
KPC: *Klebsiella pneumoniae* carbapenemase
MBL: metallo-*β*-lactamase
mCIM: modified carbapenem inactivation method
MHA: Mueller–Hinton agar
NDM: New Delhi metallo-*β*-lactamase
RT-PCR: real time-polymerase chain reaction
TSB: trypticase soy broth
VIM: Verona integron-encoded MBL
